# Retrospective analysis of MV–kV imaging‐based fiducial tracking in prostate SBRT treatment

**DOI:** 10.1002/acm2.13593

**Published:** 2022-03-26

**Authors:** Kevin Crotteau, Wei Lu, Sean Berry, Laura Happersett, Sarah Burleson, Weixing Cai

**Affiliations:** ^1^ Department of Medical Physics Memorial Sloan Kettering Cancer Center New York New York USA; ^2^ Department of Radiation Oncology Northwell Health New Hyde Park New York USA

**Keywords:** fiducial tracking, motion tracking, MV–kV, prostate SBRT

## Abstract

**Purpose:**

Motion management is critical for prostate stereotactic body radiotherapy (SBRT) due to its high fractional dose and proximity to organs at risk. This study seeks to quantify the advantages of MV–kV tracking over kV imaging alone through a retrospective analysis of over 300 patients who underwent prostate SBRT treatment using MV–kV tracking.

**Methods:**

An MV–kV imaging‐based fiducial tracking technique has been developed at our institute and become a standard clinical practice. This technique calculates three‐dimensional (3D) fiducial displacement in real time from orthogonal kV and MV images acquired simultaneously. The patient will be repositioned if for two consecutive MV–kV data points, the motion is larger than a tolerance of 1.5 mm in any of the lateral, superior–inferior, and/or anterior–posterior directions. This study retrospectively analyzed detected 3D motions using an MV–kV approach of 324 patients who recently underwent prostate SBRT treatments. An algorithm was developed to recover the 2D motion components as if they were detected by kV or MV imaging alone.

**Results:**

Our results indicated that out‐of‐tolerance motions were primarily limited to the range of 1.5–3 mm (>95%). The motions are primarily anterior–posterior and superior–inferior, with less than 14.8% of the occurrences in the lateral direction. Compared to out‐of‐tolerance occurrences detected by MV–kV approach, kV alone caught 46.6% of motions in all three directions, and MV alone caught 46.7%. kV alone shows an overall missing rate of 45.8% for superior–inferior motions and 38.6% for lateral motions. It is also demonstrated that the detectability of motion in specific directions greatly depends on gantry angles, as does the missing rate.

**Conclusions:**

Our study demonstrated that MV–kV imaging‐based intrafraction motion tracking is superior to single kV imaging for prostate SBRT in clinical practice.

## 1 INTRODUCTION

Hypofractionation and ultra‐hypofractionation using stereotactic body radiotherapy (SBRT) are becoming the preferred treatment methods for patients with low‐ to intermediate‐risk prostate cancer.^1^, ^2^ SBRT's advantages include decreased treatment time, increased survival rate, and increased cost‐effectiveness^2^ while demonstrating a limited increase in acute toxicity when delivery is precise and accurate.^3^ Intratreatment motion management is critical to improve treatment accuracy and decrease side effects. Traditional conformal radiation therapy achieves this through pretreatment alignment, where the patient's isocenter is roughly aligned using external markers. After, a kV pair or portal image is taken and registered with planning DRRs for fine‐tuning. While this can be sufficient for sites with limited motion, some movement cannot be immobilized, such as breathing, rectum filling, and bladder filling, which can displace the treatment target and tissues. The prostate is a site that is significantly affected by such motion between and during fractions.^4^ This motion enlarges the internal margins needed to ensure sufficient coverage, presenting an increased risk of acute toxicity for the radiosensitive tissues surrounding the prostate.^5^, ^6^, ^7^

Over the years, clinicians have developed new techniques to manage intratreatment motion. One technique is to implant radiofrequency beacons, such as Varian Medical System's (Palo Alto, CA) Calypso, to provide real‐time localization.^8^, ^9^, ^10^ While this approach does not add any additional dose, it suffers from a patient size limit due to signal attenuation, a costly separate system, 1 mm minimum resolution,^11^ and MR artifacts limiting imaging post implantation.^12^ Clinics also use bony landmarks or implanted radiopaque fiducials with onboard imaging to monitor motion. This is implemented through triggered kV images, such as Varian's Intrafraction Motion Review (IMR), which displays fiducial contours retrieved from the treatment planning scan against fiducial projections on kV images acquired during treatment. This offers a more streamlined solution, as it is readily integrated on modern linear accelerators and has lower costs and better localization.^13^, ^14^ However, on‐board kV imaging cannot image motion along the kV beam direction and may miss dosimetrically significant motions.

To overcome these limitations, investigators started adding MV imaging to develop MV–kV imaging systems.^15^, ^16^, ^17^, ^18^, ^19^, ^20^, ^21^ As MV and kV images are acquired from orthogonal directions, they provide complementary information to localize motion in three dimensions (3D). Our institution developed a fiducial tracking technique using simultaneous MV and kV images for prostate SBRT treatment. This technique accurately calculates 3D fiducial displacement from orthogonal kV and MV images acquired by standard gantry‐mounted onboard imaging systems.^13^, ^14^, ^22^, ^23^ In‐house software automatically identifies fiducials in MV and kV images, registers them to template images in the “ideal” fiducial position derived from the treatment plan, and generates the central position of the fiducials in 3D. Our institution began implementing this approach clinically in 2016, and it is now the standard clinical routine for prostate SBRT treatment.

This study seeks to quantify the advantages of MV–kV tracking over kV imaging alone through a retrospective analysis of over 300 patients who underwent prostate SBRT treatment using MV–kV tracking.

## 2 MATERIALS AND METHODS

### 2.1 Treatment planning

**FIGURE 1:**
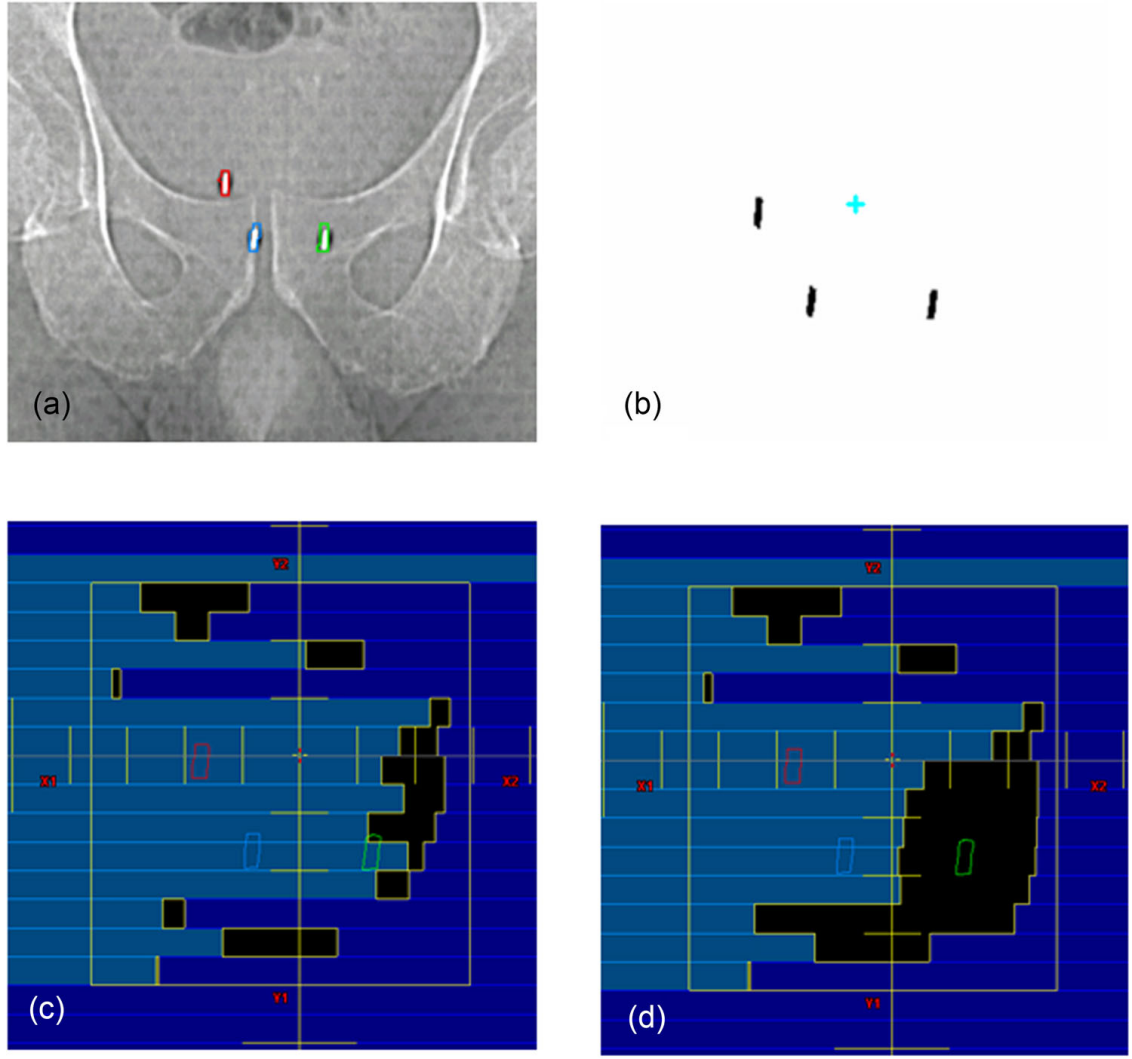
Fiducials prepared for the MV–kV treatment. (a) CT scouts showing three fiducials, (b) fiducial templates, (c) multi‐leaf collimator (MLC) aperture before modification, and (d) MLC aperture after modification

All patients first underwent transrectal ultrasound‐guided implantation of three gold cylindrical fiducials (5 × 1.2 mm) into the base, mid gland, and apex of the prostate.^22^ Patients were instructed to drink four cups of water approximately 30 min prior to simulation and all treatments (to develop a full bladder), and were positioned supine on the couch and immobilized using a customized aqua‐plastic mold. The prostate, seminal vesicles, fiducials, and organs at risk (OARs) were contoured using MR scans. A VMAT plan consisting of two full arcs was optimized, and a 3D dose was calculated on a synthetic CT computed from an MR water sequence. Multi‐leaf collimator (MLC) apertures were then modified at the control points at 20° gantry angle intervals to ensure at least one fiducial is visible through the MLC aperture.^23^ The modification increased the dose to the PTV by less than 0.7%^23^ and up to 0.5% for OARs, which is accounted for in the planning procedure. Figure 1a shows the three implanted fiducials in a CT scout image contoured in red, blue, and green, compared to fiducial templates in Figure 1b. Figure 1c shows the original MLC aperture at a control point, and Figure 1d shows the modified aperture where some MLC leaves are opened to expose at least one fiducial.

### 2.2 MV–kV tracking

Fiducial templates were generated based on projections of fiducial contours at all gantry angles with 1° intervals. Thus, the templates served as “ground truth” for patient setup and monitoring during treatment. A CBCT scan confirmed proper bladder and rectum filling for setup. If noticeable rotational misalignment (pitch, roll, and yaw) was observed, CBCT was used to minimize rotational errors. Then SequenceReg acquired a kV image pair, registered fiducials in templates to their counterparts in kV images, and set the center of fiducials as the zero position for tracking.

**FIGURE 2:**
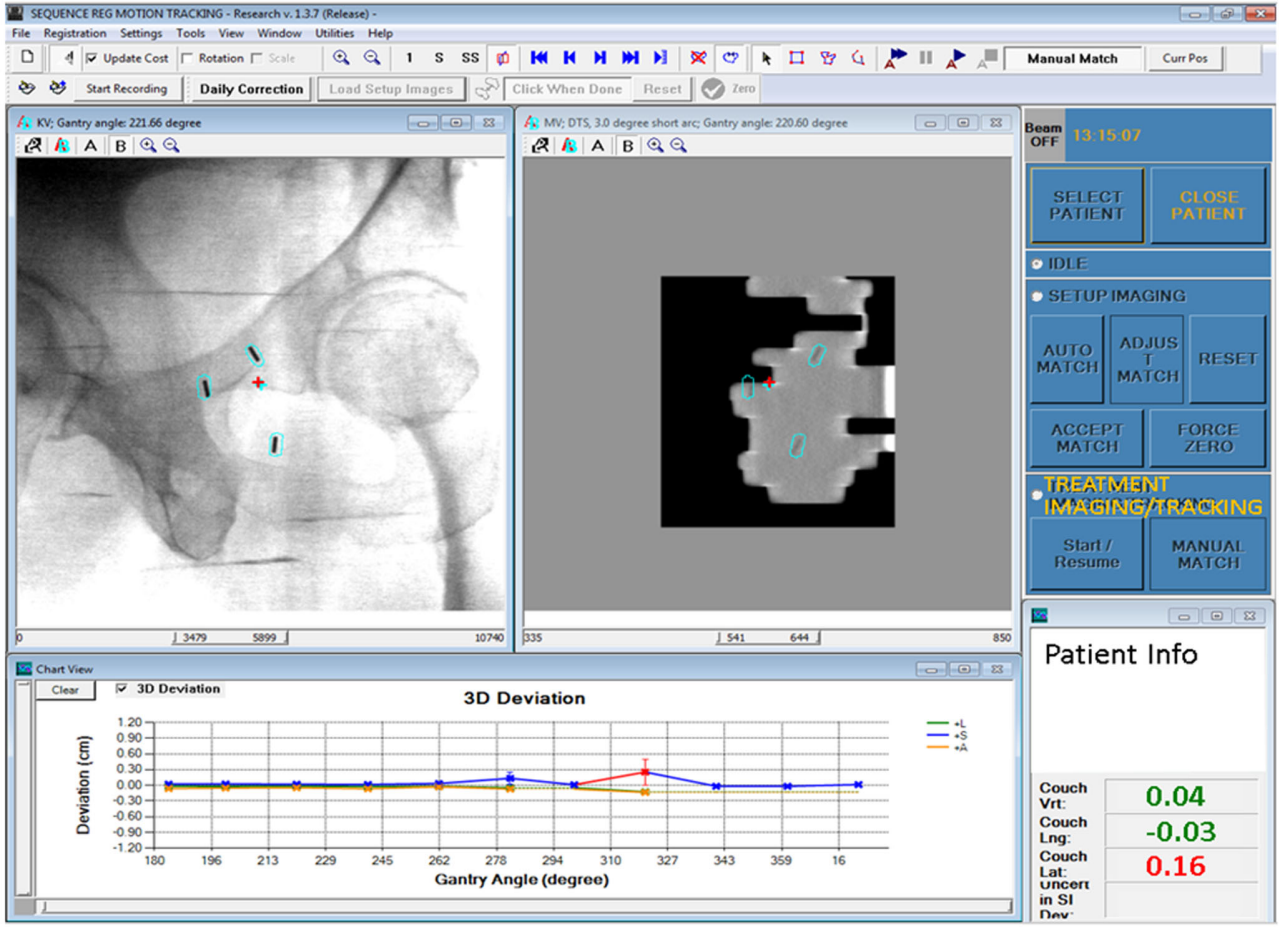
SequenceReg user interface showing template registration to MV and kV images and calculated 3D displacement at the lower right corner

During the treatment delivery, kV images were triggered every 20°, and simultaneously, MV images were reconstructed into Short‐Arc Digital Tomosynthesis (SA‐MVDTS) images that better manifest the quality of fiducials.^13^ An in‐house tracking platform named SequenceReg captured MV and kV images, identified fiducials on MV and kV images, registered fiducials to templates and quantified 3D motion as lateral, superior–inferior, and anterior–posterior shifts. Figure 2 shows the software interface. Therapists were alerted if the fiducial position deviated by more than a defined tolerance of 1.5 mm in any direction for at least two 20° arcs, and the patient would be repositioned. SequenceReg recorded the date, time, positional data, deviation, gantry angle, and tracking data after each successful treatment. Fault values were generated within the record due to pauses in treatment for repositioning or poor auto‐matching.

### 2.3 Recovering 2D information

**FIGURE 3:**
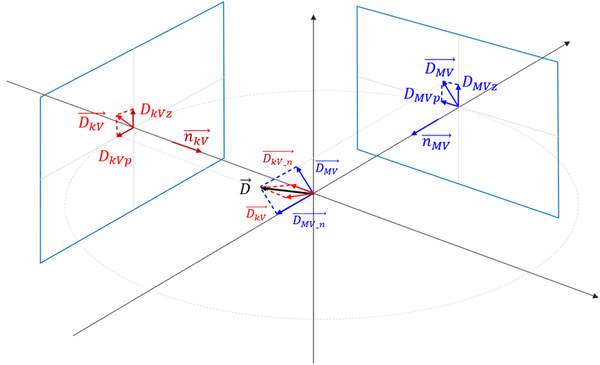
Geometry to recover 2D motion components (from recorded 3D motion) as if they were detected by either MV or kV imaging alone

SequenceReg automatically calculates the 3D fiducial motion but does not save the intermediate 2D motion from either kV or MV images. We developed an algorithm that was able to recover the 2D motion components as if they were detected by kV or MV imaging alone. Figure 3 shows the geometry of motion vector components in the room coordinate system at an arbitrary gantry angle.

MV imaging alone can identify motion in two directions on the MV detector plane, $\vec{D_{\mathrm{MV}p}}$ and $\vec{D_{\mathrm{MV}z}}$, where the $\vec{D_{\mathrm{MV}z}}$ component is the direction parallel to the anterior–posterior direction (${\vec{n}}_{z}$) and the $\vec{D_{\mathrm{MV}p}}$ is in the perpendicular direction on the MV detector (${\vec{n}}_{p}$). Given the 3D motion vector $\vec{D}$ as provided by SequenceReg, the component of $\vec{D}$ that can be detected by the MV detector is $\vec{D_{\mathrm{MV}}}$, which is parallel to the detector plane, and the component that cannot be detected is $\vec{D_{MV_{n}}}$, which is normal to the detector plane. Given ${\vec{n}}_{\mathrm{MV}}$, the normal vector of the MV detector plane, we have

(1) $$\vec{D_{MV_{n}}}=\left(\vec{D}\cdot {\vec{n}}_{\mathrm{MV}}\right)\;{\vec{n}}_{\mathrm{MV}}$$

(2) $$\vec{D_{\mathrm{MV}}}=\vec{D}\;-\vec{D_{MV_{n}}}$$

Now we can decompose $\vec{D_{\mathrm{MV}}}$ into $D_{\mathrm{MV}p}$and $D_{\mathrm{MV}z}$ using Equations (3) and (4):

(3) $$D_{\mathrm{MV}p}=\vec{D_{\mathrm{MV}}}\;\cdot {\vec{n}}_{p}$$

(4) $$D_{\mathrm{MV}z}=\vec{D_{\mathrm{MV}}}\;\cdot {\vec{n}}_{z}$$

All the directional vectors are determined by gantry angle. To calculate the kV components $D_{\mathrm{kV}p}$ and $D_{\mathrm{kV}z}$, the formulation is the same, except the angle is kV imaging angle. Thus, the *Z* components of the MV and kV coordinate system correspond to anterior–posterior motion, while the *P* component is a mixture of superior–inferior and lateral (left–right) motions, depending on gantry angle.

### 2.4 Patient data analysis

Statistics of motion was analyzed using the 3D components (lateral, superior–inferior, and anterior–posterior) and decomposed 2D components in the kV and MV imaging planes. Some of the occurrences of large motions (>5 mm) likely result from failures of matching. In clinical practice, therapists visually monitored the template matching in both MV and kV images to make sure auto‐matchings were reasonable and thus derived 3D shifts were reliable.

To compare to the performance of Varian's IMR, 2D kV components were examined to investigate how kV alone would perform in detecting the motions. For example, kV at gantry zero cannot detect superior–inferior motions but can accurately detect lateral motions, that is, the capability of motion detection depends on both gantry angle and motion direction. Here, a metric “detectability” is defined to describe the minimum patient motion in either the lateral or superior–inferior direction that 2D kV imaging can detect as the threshold value (1.5 mm for the study). For example, when the kV imaging angle is at 0°, the detectability of lateral motion is 1.5 mm as kV can capture the entire lateral motion. When kV imaging angle is at 60°, a lateral motion of 3 mm or larger shows a motion of 1.5 mm (3 mm × cos(60°) = 1.5 mm, the threshold value) or larger in the detector plane, so the detectability of lateral motion at kV angle 60° is 3 mm. This means any lateral motion less than 3 mm in magnitude will not be detected as out of tolerance. Now if there is a lateral motion of 2.0 mm, it will be detected as 1.0 mm (2 mm × cos(60°) = 1 mm) in the detector plane, which will be considered as within tolerance, as 1.0 mm < 1.5 mm because the actual shift of 2.0 mm is less than the detectability of 3 mm. In general, the detectability is defined as

(5) $$\mathrm{Detectabilit}y_{\mathrm{superior}-\mathrm{inferior}}=\;\mathrm{threshold}*\frac{1}{\sin\left(\alpha \right)}$$

(6) $$\mathrm{Detectabilit}y_{\mathrm{lateral}}=\;\mathrm{threshold}*\frac{1}{\cos\left(\alpha \right)}$$

where α is kV imaging angle. Any motion smaller than the detectability for that angle will be missed by 2D kV motion tracking. Thus, we can calculate the missing rate by comparing the out‐of‐tolerance cases with 3D tracking and out‐of‐detectability cases in 2D kV tracking.

## 3 RESULTS

From May 2019 to May 2020, 324 patients received prostate SBRT treatment using MV–kV intrafraction fiducial tracking at our institution. There was a total of 52 752 MV–kV pairs for analysis. On average, there were approximately 1.7 treatment interruptions per patient for repositioning due to motion. Total 134 of 324 patients (41.4%) experienced repositioning at least once.

**FIGURE 4:**
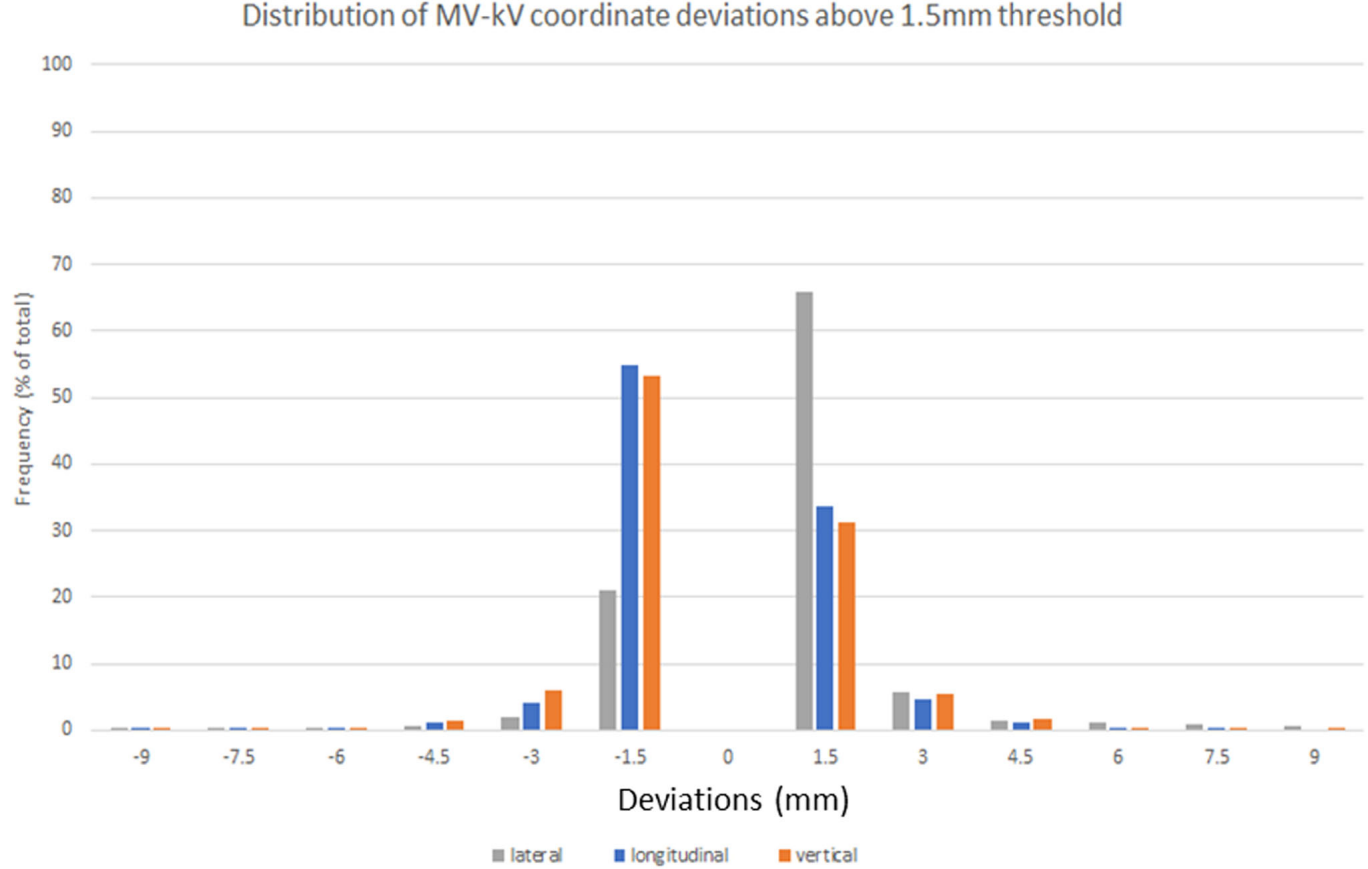
Out‐of‐tolerance values sorted by magnitude into 1.5 mm bins for the MV–kV lateral, anterior–posterior, and superior–inferior coordinates

Table 1 compares out‐of‐tolerance occurrences reported by the 3D MV–kV approach and occurrences that could be detected by the 2D approach alone (kV or MV). Out of the total of 52 752 MV–kV pairs, 45 050 (85.3%) pairs were within the ±1.5 mm tolerance. We can see from Table 1 that ≥95% of the out‐of‐tolerance shifts are within 1.5–5 mm range. The magnitude of the motion is sorted into histograms in Figure 4 with 1.5 mm bin sizes. As we only considered points beyond the ±1.5 mm tolerance, bins from −1.5 to 1.5 mm are empty.

**TABLE 1:** Statistics of out‐of‐tolerance occurrences

	**1.5–3.0 (mm)**	**3.0–5.0 (mm)**	**Total: 1.5–5.0 (mm)**
*Z*_MV_ and *Z*_kV_	2592 of 2696 (96.1%)	87 of 2696 (3.3%)	2679 of 2696 (99.4%)
*P*_MV_	869 of 903 (96.2%)	27 of 903 (3%)	896 of 903 (99.2%)
*P*_kV_	863 of 897 (96.2%)	27 of 897 (3.1%)	890 of 897 (99.3%)
Unique 3D MV–kV	7324 of 7702 (95.1%)	201 of 7702 (2.6%)	7525 of 7702 (97.7%)
Lateral MV–kV	1982 of 2280 (86.9%)	192 of 2280 (8.5%)	2174 of 2280 (95.4%)
Superior–inferior MV–kV	5640 of 6464 (87.3%)	724 of 6464 (11.2%)	6364 of 6464 (98.5%)
Anterior–posterior MV–kV	5720 of 6680 (85.6%)	874 of 6680 (13.1%)	6594 of 6680 (98.7%)

*Note*: *Z*_MV_ and *Z*_kV_ represent the posterior and anterior motions, *P*_MV_ represents lateral motion, and *P*_kV_ represents superior–inferior motion (all at gantry angle 0).

Our data indicated 7702 occurrences (14.7% out of 52 752 pairs) where the prostate was above the threshold, while the kV imaging alone caught 3593 occurrences, and MV imaging caught a comparable number of 3599 (Table 2). With both MV and kV imagers sharing a *Z* coordinate, each imager's respective *P* coordinates were of interest. For the kV, we recorded 897 instances, and for the MV, 903 instances were above the 1.5 mm threshold for two or more 20° arcs. Thus, we can see that while they both catch a similar amount of displacement, they are not nearly as effective as combination 3D MV–kV imaging, which detected 7702 unique coordinates above the action threshold. The term unique is used as our methodology counted multiple displacements for the same MV–kV pair as a single out‐of‐tolerance warning. For example, if the deviation in the *X*, *Y*, and *Z* were 1.7, 0.5, and −2.0 mm, respectively, it would be a single warning for our system. Additionally, we decomposed the displacement for each axis above 1.5 mm, showing 85.2% of displacements involved a superior–inferior and anterior–posterior component, and over half the displacements above the threshold involved motion on two axes. In Figure 4, the histogram shows the displacement for each coordinate and duplicates detected by both planes.

**TABLE 2:** Out‐of‐tolerance occurrences detected by MV and kV alone

	**MV**	**kV**	**MV–kV**
1.5–3.0 (mm)	3461	3455	7324
3.0–5.0 (mm)	114	114	201
Total occurrences above 1.5 (mm)	3599	3593	7702

We extracted the out‐of‐tolerance lateral and superior–inferior occurrences from the 3D coordinates and compared their magnitude to the imaging angle‐dependent detectability defined in Section 2.4. The 3340 ${\vec{n}}_{z}$ components, which are in the anterior–posterior direction, can always be detected with only kV imaging, and therefore the detectability in anterior–posterior direction is 1.5 mm for all gantry angles.

**FIGURE 5:**
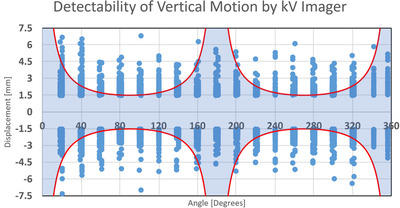
Magnitude of displacement for anterior–posterior motion on MV–kV imaging system against gantry angle (blue) and detectability threshold of kV‐only imaging (red)

**FIGURE 6:**
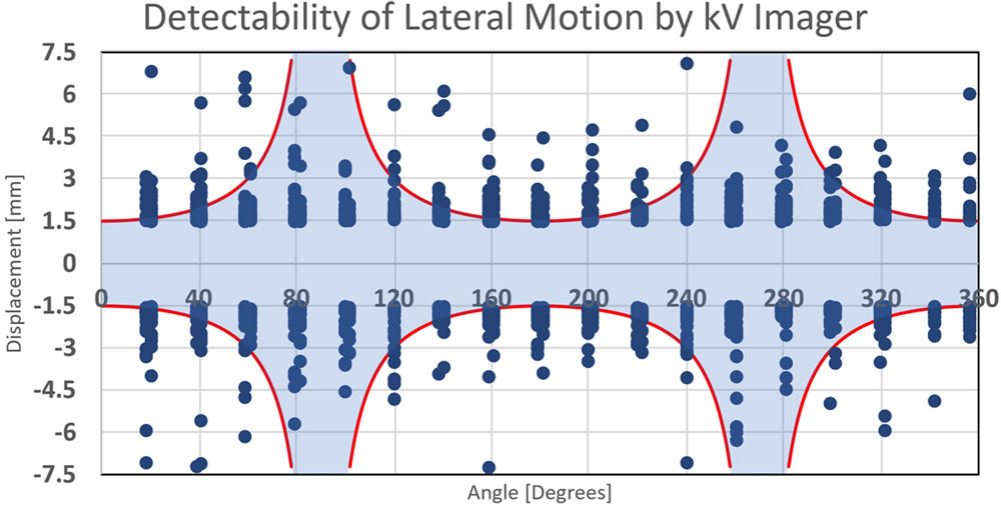
Magnitude of displacement for left–right motion on MV–kV imaging system against gantry angle (blue) and detectability threshold of kV‐only imaging (red)

**FIGURE 7:**
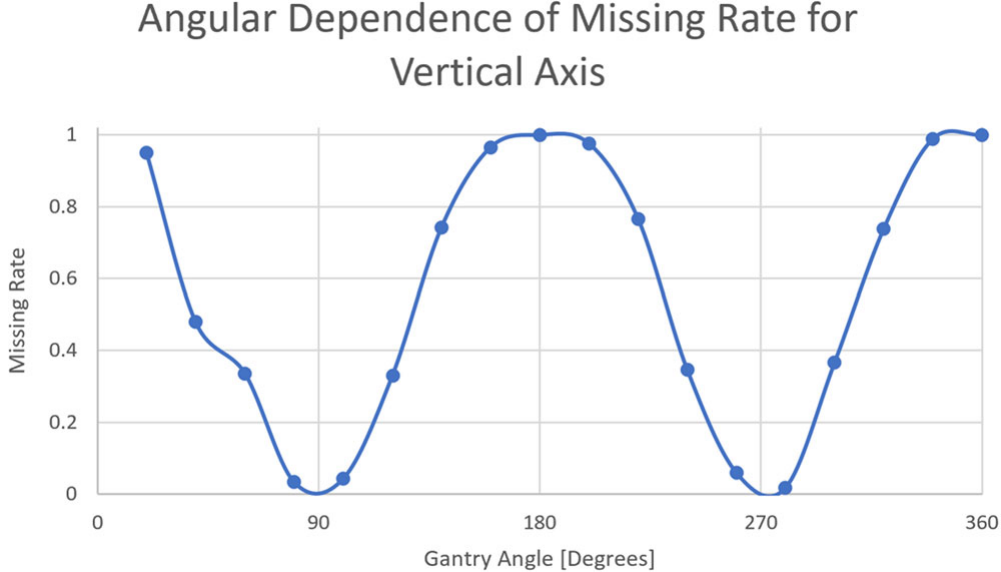
Angular dependence of missing rate for 2D kV imaging‐based detection of anterior–posterior movement

**FIGURE 8:**
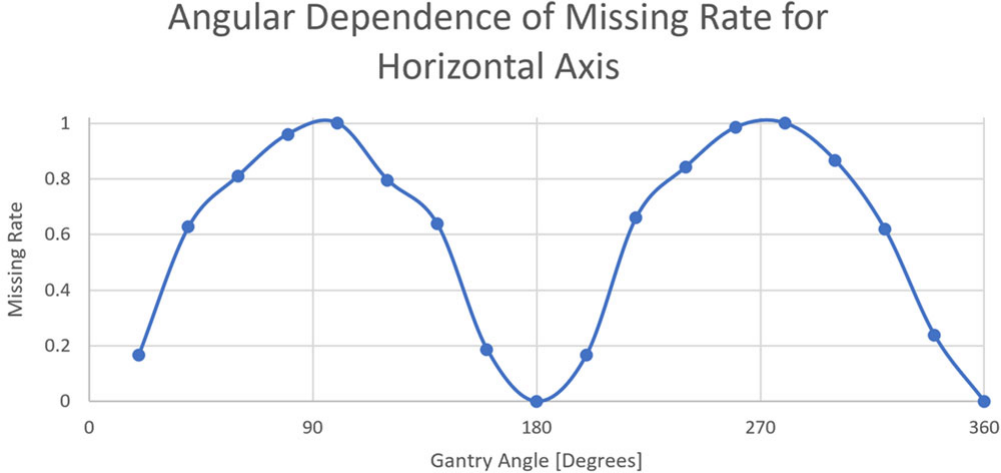
Angular dependence of missing rate for 2D kV imaging‐based detection of left–right movement

With the threshold for 3D motion set at 1.5 mm, we plotted the magnitude of the out‐of‐tolerance lateral and superior–inferior components for the 3D MV–kV imaging against kV imaging angle in Figures 5 and 6. Red curves show detectability in superior–inferior and lateral directions, respectively, in the two figures. Points in the shaded area represent occurrences missed by 2D kV imaging alone. As our kV pairs are triggered every 20°, they are distributed in columns 20° apart. Overall, 2D kV tracking had a 38.6% miss rate for lateral motions and a 45.8% miss rate for superior–inferior motions. This is also summarized in Table 3. The angular dependence of the missing rate for lateral and superior–inferior is plotted in Figures 7 and 8, respectively. At each triggered imaging angle, the missing rate is the ratio of missed 2D kV imaging out‐of‐tolerance points (shaded area) against the total out‐of‐tolerance points detected by 3D MV–kV at that kV imaging angle.

**TABLE 3:** Missing rate of 2D kV imaging (tolerance of 1.5 mm)

	**Superior–inferior motion**	**Lateral motion**
Total out‐of‐tolerance points	3232	1130
Number of misses	1479	436
Missing rate	45.8%	38.6%

## 4 DISCUSSION

Our results indicated that out‐of‐tolerance motions were primarily limited to the range of 1.5–3 mm (>95%). The motions are primarily anterior–posterior and superior–inferior, with less than 14.8% of the occurrences in the lateral direction. These shifts are most likely related to bladder or rectum filling. The average number of interruptions for patient repositioning was 1.7 per patient. This result generally agreed with a preliminary clinical research reported by Gorovets et al.^22^ that demonstrated the accuracy of MV–kV technique. With a tolerance of 1.5–2 mm, they reported that the median number of interruptions for patient repositioning was one per patient. It is reasonable that our tighter threshold requires more interruptions. Levin‐Epstein et al.^12^ utilized orthogonal kV pairs to assess prostate displacement and reported that about 98% and 95% of patients experienced motion of 3 mm or less in SI and AP directions, respectively, which generally agrees with our numbers of 98.6% (52 082 out of 52 752) and 98.3% (52 028 out of 52 752).

Compared to out‐of‐tolerance occurrences detected by MV–kV approach, kV alone caught 46.6% of motions in all three directions, and MV alone caught 46.7%. It is clear that kV or MV alone has the same detectability as that of MV–kV approach for anterior–posterior motion and there should be no missing occurrences. However, kV alone shows a missing rate of 45.8% for superior–inferior motions and 38.6% for lateral motions. The high miss rate demonstrates the need for 3D MV–kV tracking. It is also demonstrated that the detectability of motion in specific directions greatly depends on gantry angles (and thus imaging angles), as does the missing rate. The kV image would miss all superior–inferior motions at angles 0° and 180°, and for all lateral motions at angles 90° and 270°. However, 3D MV–kV would still catch them all.

Fiducial migration was observed occasionally during patient setup. In such a scenario, the patient was aligned using anatomic information instead of fiducials. The in‐house software then separately registered each fiducial in templates to its counterpart in kV images and set the center of the three fiducials as the new zero position for tracking. The same threshold was used for tracking the motion.

There are several limitations in the development and maintenance of 3D MV–kV imaging that warrant consideration. The MV–kV approach relies on the accuracy of automatic matching of fiducial templates to both MV and kV images. The auto‐matching technique might fail if the image quality is low or fiducial positions are too close. In this study, some data points with large motions (>5 mm) might be caused by failures in auto‐matching. The in‐house software must be customized and interfaced with the existing treatment process, and a quality assurance program must also be designed to ensure the accuracy and robustness of the MV–kV procedure. In clinical practice, therapists must also be trained and credentialed to operate the MV–kV system. As a safeguard, they were instructed to visually verify the quality of fiducial matching on a second monitor. They are expected to ignore the 3D shift results if the auto‐matching is not reasonable. All these considerations require extra resources to implement the MV–kV tracking technique. Nevertheless, these limitations do not impact the statistical significance of our results.

## 5 CONCLUSION

Our study demonstrated that MV–kV imaging‐based intrafraction motion tracking is superior to single kV imaging for prostate SBRT in clinical practice. Effective motion management is critical for improving treatment accuracy and improving our ability to limit damage to surrounding tissues. Our next step is to evaluate the clinical effects of our technique by helping physicians investigate prostate SBRT treatment outcomes with MV–kV as a motion tracking method.

## AUTHOR CONTRIBUTIONS

Kevin Crotteau, Weixing Cai, Wei Lu, and Sean Berry contributed to the design and implementation of the research, to the analysis of the results, and to the writing of the manuscript. Laura Happersett and Sarah Burleson provided help in study design and preparing patient data.

## CONFLICT OF INTEREST

The authors declare no conflict of interest.
